# Factors that influence the characteristics of needles and syringes used by people who inject drugs in Tajikistan

**DOI:** 10.1186/s12954-015-0069-7

**Published:** 2015-10-16

**Authors:** William A. Zule, Alisher Latypov, David Otiashvili, Irma Kirtadze, Umedjon Ibragimov, Georgiy V. Bobashev

**Affiliations:** RTI International, 3040 Cornwallis Road, Research Triangle, Park, NC 27709-2194 USA; Management Sciences for Health, Leadership, Management and Governance, Kiev, Ukraine; Addiction Research Center, Alternative Georgia, Tbilisi, Republic of Georgia; Business School, Ilia State University, Tbilisi, Republic of Georgia; Behavioral Sciences and Health Education Department, Emory University, Rollins School of Public Health, Atlanta, Georgia USA

**Keywords:** Low dead space syringes and needles, People who inject drugs, HIV transmission, Tajikistan

## Abstract

**Background:**

“Low dead space” syringes with permanently attached needles retain less fluid, blood, and HIV after use than standard “high dead space” syringes. This reduces the probability of HIV transmission if they are shared by people who inject drugs (PWID). The World Health Organization recently recommended that needle and syringe programs (NSP) offer clients low dead space syringes. The success of this recommendation will depend on PWID switching to low dead space needles and syringes. This paper examines the needles and syringes that PWID in Tajikistan use and factors that influence their choices.

**Methods:**

In May 2014, we conducted six focus groups in Kulob and six in Khorog, Tajikistan, with a total of 100 participants. NSP staff members recruited participants. Focus group topics included the needles and syringes used and factors that influence choice of needles and syringes. Focus groups were conducted in Russian and Tajik, audio recorded, transcribed, and translated into English. The translated files were imported into NVivo 10 for coding and analysis.

**Results:**

All participants in both cities were male and reported injecting heroin. Everyone also reported using syringes with detachable needles almost exclusively. The most popular syringe sizes were 2 and 5 ml. Needles ranged in gauge from 25 to 21 g. Needle gauge was influenced by the size of the vein, the viscosity of drug solution to be injected, and problems with blood clotting. Needles ranged in length from 12 to 38 mm, with 25 and 32 mm being the most popular. Needle length was influenced by the depth of the vein being used. Many PWID inject volumes of fluid greater than 1 ml into deep veins that require needles at least 25 mm long and 25 g in diameter.

**Conclusion:**

Most low dead space syringes are 1-ml insulin syringes with 12 mm 28 g permanently attached needles. Findings from this project suggest that these will not be acceptable to PWID who need larger syringes and longer and thicker needles that are detachable. Low dead space detachable needles appear to be an acceptable option that could overcome barriers to the widespread use of low dead space equipment for reducing HIV and HCV transmission.

## Background

HIV and hepatitis C virus (HCV) are serious problems in Tajikistan and other Central Asian Republics among people who inject drugs (PWID) [1, 2]. Most HIV and HCV infections among PWID are attributed to direct needle and syringe sharing (i.e., multi-person use of a needle and or syringe) [3, 4] or indirect sharing (i.e., sharing drug preparation materials or the liquefied drug solution) [5, 6]. PWID use a variety of different needle and syringe combinations [7]. Some needles and syringes retain visibly more fluid and blood (i.e., “high dead space” syringes) after they have been used than other needle and syringe combinations retain [8, 9]. Syringes that retain less blood have been referred to as “low dead space” syringes [10, 11]. In laboratory tests, HIV and HCV survived longer in high dead space syringes than in low dead space syringes [12, 13]. Evidence from a variety of sources suggests that the probability of HIV and HCV transmission associated with exposures that involve high dead space needles and syringes is greater than the probability of transmission associated with exposures involving low dead space needles and syringes [11]. This raises the possibility that getting PWID to use only low dead space needles and syringes could reduce injection-related HIV and HCV transmission even if they continued to engage in unsafe injecting practices at the same rate [14]. Circumstantial evidence in support of this observation led the World Health Organization (WHO) to include a recommendation for needle and syringe programs to offer their clients low dead space syringes [15]. The impact of this recommendation will depend on the willingness of PWID to use only low dead space needles and syringes [10].

**Fig. 1:**
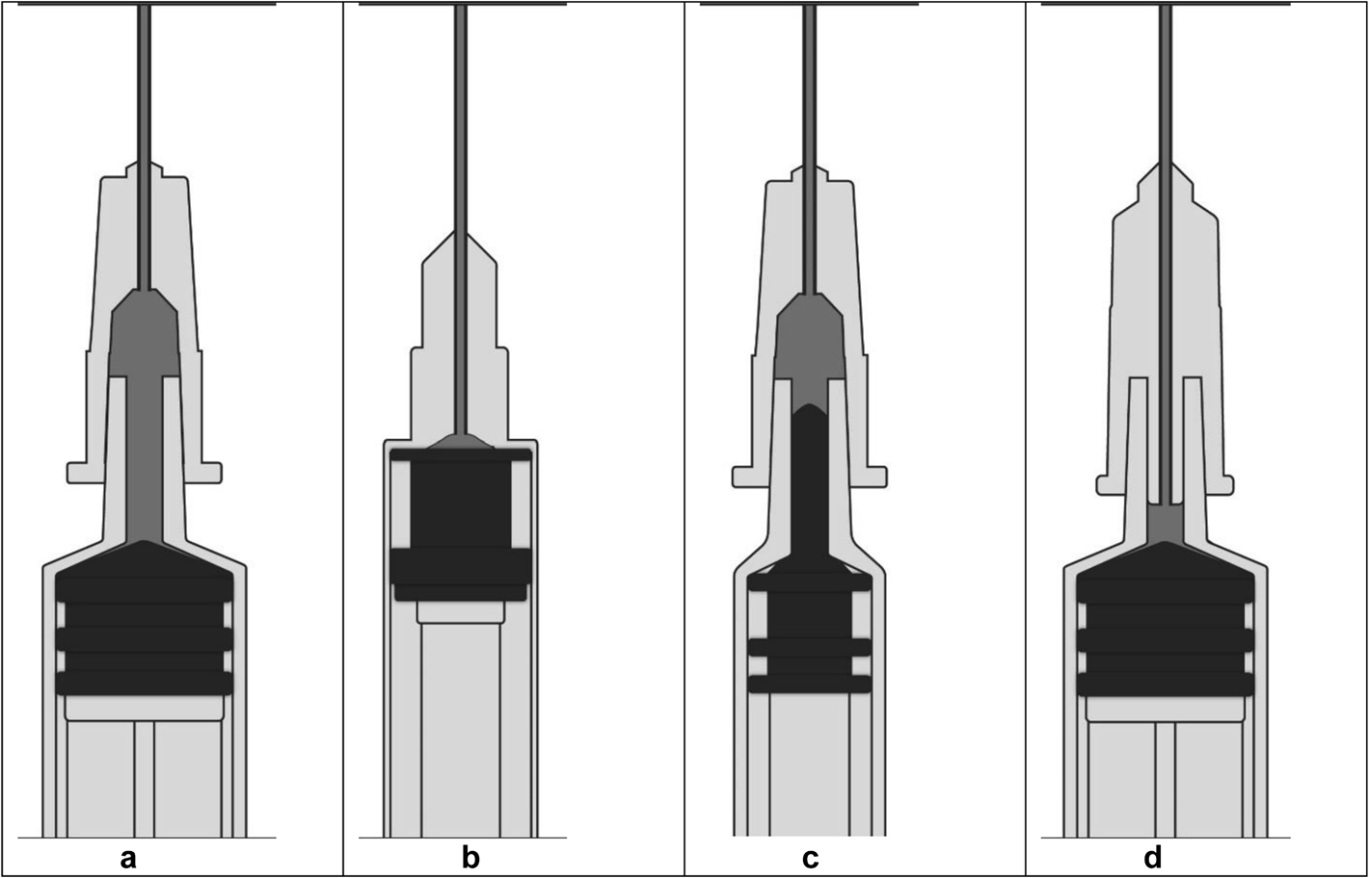
Cross-sectional view illustrating dead space in standard needles and syringes and low dead space options. **a** Standard needle on standard syringe; **b** Low dead space syringe with permanently attached needle; **c** Standard needle on low dead space syringe; **d** Low dead space needle on standard syringe

Figure 1a illustrates the dead space in a standard needle and syringe. The first low dead space syringes, which were insulin syringes, had a permanently attached needle that extended through the nozzle of the syringe to the base of the barrel (Fig. 1b). To this day, virtually all syringes with permanently attached needles are low dead space. Until relatively recently, syringes with detachable needles have all been high dead space. However, this situation is changing. Some syringes have an elongated tip on the plunger that extends into the nozzle of the syringe (Fig. 1c). This eliminates almost all of the dead space in the nozzle of the syringe; however, it does not affect the dead space in the hub of the needle. Some needles are also designed to reduce dead space. These needles have a spike that extends into the nozzle of the syringe (Fig. 1d). These needles can eliminate most of the dead space in the hub of the needle and in the nozzle of the syringe. The actual reduction in dead space achieved by these low dead space detachable needles varies from syringe to syringe. This is because there are no standards for the wall thickness and height of syringe nozzles.

The characteristics of needles and syringes are likely to influence the probability of HIV and HCV transmission if they are shared. Nevertheless, relatively few studies of PWID have examined the characteristics of the needles and syringes that PWID use or the factors that influence their choice [7, 16]. These few studies indicate that PWID use needles that vary in length and gauge (i.e., outside diameter). The syringes that PWID use vary in barrel capacity and in whether the needles are permanently attached or detachable. In addition to the design (i.e., high dead space or low dead space), syringe barrel capacity, needle length, and needle gauge all influence dead space volume in a needle and syringe combination [17].

This paper explores the factors that influence the length and gauge of needles, the barrel capacity and design (i.e., fixed needle, detachable needle) of syringes that PWID in Tajikistan prefer and use. The paper also examines the interrelationships among these factors and their implications for efforts to transition PWID from high dead space to low dead space needles and syringes.

## Methods

This paper reports findings from focus groups that were conducted during the formative phase of a project to develop and pilot test an intervention to transition NSP clients from standard (high dead space) needles and syringes to low dead space needles and syringes. We selected Tajikistan based on findings from a previous project. In that project, NSP in 70 cities and 30 countries across Europe and Asia completed a brief survey regarding local HIV prevalence among PWID, the main drugs injected locally, the characteristics of the needles and syringes that they distributed, and several other issues [11, 17].

### Recruitment and eligibility

In May 2014, we conducted 12 focus groups (6 in Kulob and 6 in Khorog) with a total of 100 participants (47 in Khorog and 53 in Kulob). Participants were recruited by needle and syringe program (NSP) staff members either at the NSP offices or while doing outreach. The focus groups were conducted in the NSP office in each city. Eligibility criteria included a minimum age of 18 years, self-reported injection in the previous 30 days, obtaining syringes from the NSP at least two times in the last 30 days, and the ability to speak Tajik or Russian.

### Focus group procedures

The focus groups were conducted by two trained facilitators and attended by two observers (the Principal Investigator and the local Project Coordinator). The focus group guide explored the following topics in detail: substance use patterns, injecting drug use practices, types of needles and syringes used, reasons for using specific types of needles and syringes and their combinations, syringe cleaning procedures, sources of injecting equipment. Each focus group lasted between 60 and 90 min, was digitally recorded and transcribed directly into native language. All transcripts were translated into English to permit review and discussion with US collaborators.

The study received ethical approval from the Committee of Medical Ethics of Tajikistan and the RTI International’s Office of Research Protection. All participants provided oral informed consent, and received the equivalent of $5 US for their participation.

### Analysis

NVivo v.10 (http://www.qsrinternational.com/products_nvivo-mac.aspx) was used for the qualitative content and thematic analysis. The transcripts were not back-translated, since one of the coders (also one of the co-authors), fluent in Russian, Tajik, and English languages, constantly compared English translation with the original version. In addition, another co-author, also fluent in all three languages, reviewed translation and analysis findings to ensure they adequately reflected the original language transcription. Coders were two individuals experienced in qualitative research and familiar with the drug scene and overall cultural context of Tajikistan. Coders read and reread the transcripts and developed a draft code book with major categories, sub-categories, and codes based on the topics in the FGD guide. To ensure consistency and similarity of coding, each coder independently coded two transcripts (one per study site), then compared, discussed, and revised codes and the code book. Further, each coder independently coded all remaining transcripts. Coders iteratively communicated and discussed all revisions to the code book, including addition of new codes, grouping and regrouping of codes and categories in the coding tree until an agreement was reached. After completion of the coding process, inter-rater reliability coefficient was calculated. Once a certain code had been created in NVivo, it was then applied in relevant places in other interviews. Thus, we had a set of nodes (codes), with reference and source numbers. During reading of transcripts, notes, memos and annotations were done; then, linkages/relationships between relevant statements were created. In the final stage, the two independent sets of codes of these coders were merged to track the contributions of different raters and to compare coding as a way of facilitating common approaches across coders. Coder Comparison Analysis provides information about code type, code, source type, source, and source folder. Two measures of agreement [18] between coders were calculated in NVivo [19]:

- ▪ *Percentage agreement* [20] is calculated from the number of units (usually characters) for which two coders are in agreement (including both presence and absence of coding for the particular node), compared to the total number of available units.
- ▪ The *Kappa coefficient* [21] is a statistical measure of the level of agreement between two coders that takes into account the amount of random (chance) agreement that could be expected to occur.

The Kappa coefficient and percentage agreement were calculated individually for each combination of nodes and sources and for all nodes together. The results of this coding comparison query were exported from NVivo as an excel spreadsheet (using the Export List command). The average agreement rate by source size (characters) and weighted Kappa coefficient for all nodes were calculated using Excel formulas resulting in 99.69 and 0.83 %, respectively. The results of separate calculations of Kappa coefficient of key nodes and percentage agreement can be seen in Table 1.

**Table 1:** Results of calculation of average Kappa coefficient and percentage agreement across sources and nodes

Node name	Kappa (weighted)	Agreement (%)
Good quality needle and syringe	0.76	99.83 %
Bad quality needle and syringe	0.88	99.91 %
Used combinations of needles and syringes	0.73	99.72 %
Overall evaluation of low dead space syringes	0.91	99.95 %

According to results of coding comparison, we would conclude that the inter-rater reliability is excellent [22], because the obtained Kappa is higher than commonly applied criteria of 0.81 [23].

### Description of the sample

All participants were male. The mean age was 42 years (median = 43 years). Participants reported injecting an average of 15 times per week (range 1–35). Most (75 %) of participants reported that the NSP was their primary source of needles and syringes. An additional 13 % reported obtaining equal percentages of needles from NSP and other sources, and 9 % reported pharmacies as their primary source of needles and syringes. Participants reported obtaining syringes from the NSP an average of 12 (range 2–30) times in the last 30 days.

## Results

### Drugs used

All participants in both cities reported injecting heroin from Afghanistan. Kulob is approximately 70 km from Afghanistan, and Khorog is situated on the Tajik side of the Panj River, which separates Afghanistan and Tajikistan. The price of a gram of heroin cited by the majority of respondents in Khorog was 50 Somoni, the equivalent of $10 US. In Kulob, the average price of heroin was 50 Somoni, approximately $10 US, a gram. However, participants reported that prices in Kulob often fluctuate, and higher purity heroin may sell for 100 Somoni ($20 US) per gram. There was a general perception that the heroin currently being sold in both cities was not as pure as the heroin that was available in previous years. This may reflect an actual decline in the quality of heroin, or their perceptions may be clouded by nostalgia. Several participants reported that the drug sellers adulterated heroin with zopiclone or diphenhydramine. Zopiclone is from the same family of drugs as Lunesta (eszopiclone) and Ambien (zolpidem). Diphenhydramine is an antihistamine, which reports indicate has been mixed with heroin and injected in other settings [24]. Participants in Kulob reported that some PWID add diphenhydramine to heroin after they purchase it to potentiate the effects of the heroin when there is a shortage.

*“If heroin is pure, it will carry you away until the morning, you won’t feel sick. But there is shortage of [good quality] heroin now, that’s why they shoot it [mixing with medicines].*” (FGD#3, Kulob)

Several participants in Khorog reported that some PWID inject “khanka” (i.e., acetylated opium). They said “khanka” is produced by adding acetic anhydride to opium. However, “khanka” injection is relatively uncommon now because heroin is readily available in Khorog. Some participants in Kulob reported injecting “synthetic heroin” in the past. One participant described the difference in the appearance of heroin and “synthetic heroin” as follows:

*“Usually heroin has no glitter, but that one glitters like glass, it is a substitute, you buy it for the same money, the price is the same.”* (FGD#1, Kulob)

They reported that this “synthetic heroin” was sold on the market several years ago and was associated with a higher risk of overdose. No one was sure of the chemical composition of this “crystal heroin.” Most participants reported that they would only buy it if regular heroin was not available.

One participant in Kulob reported that some people who inject heroin inject eye drops that contain tropicamide to relieve withdrawal symptoms if they cannot get heroin. However, no other participant mentioned it, so this practice did not appear to be widespread in Kulob or Khorog at the time these focus groups were conducted.

### Factors that influence the characteristics of needles and syringes that are used and preferred

Most of the participants differentiate the needles by length and thickness; nobody mentioned needle gauge as the way to distinguish between the needles. Many participants distinguish needles by the color of the needle hub and according to volume of the syringe the needle usually comes with (e.g., the needle of a 2-ml syringe, the needle of an insulin-type syringe etc.). Needle hubs are color coded by gauge. However, in a previous study, we found that the color coding is not consistent across all manufacturers [17].

#### Quality of needles

Participants prefer needles that are sharp and that are securely attached to the plastic hub. Needles are mass-produced, and all manufacturers occasionally produce needles that are defective. These defects include needles that are dull, bent, or poorly secured to the plastic hub when they leave the manufacturer. Most PWID prefer needles that are sharp because they are less painful, and they do less damage to veins. The proportion of defective needles from the NSP in Khorog seemed to have increased recently.

*“Well, they are blunt, expired. Before, when it started, everything was ok, beautiful, needles were clean and fine, not expired, and now, recently, they are all blunt and of low quality. OK, but if we don’t take it, then what we will shoot with?”* (FGD#8, Khorog)

Several participants reported that when some needles are reused multiple times, the connection between the needle and the needle hub fails, and the needle detaches and remains in their arm following the injection. Although this seems to be a relatively rare occurrence, it seems to make an impression on PWID when it happens as described by a participant from Khorog.

*“The needle does not break; it just comes out of there, out of the plastic thing.*

*Old ones, expired ones.”* (FGD#8, Khorog)

#### Needle length

Needle length varied depending on the injection site/vein. Medium-length (25 mm) needles were preferred by those participants who inject into cubital (i.e., forearm or elbow) veins. Some of the participants mentioned that they use 25-mm needles for injecting into the neck. Longer (32 mm) needles are used for injecting into the groin or femoral veins. Some participants reported that PWID combine 2-ml syringes with larger needles from 5- or 10-ml syringes to inject into the groin as illustrated in the following quotes.

*“Those who shoot in veins, they use 25 [mm needles], and there are people who shot into groin, that use 32 [mm].”* (FGD#10, Khorog)

*“If someone has large veins, good veins, he would use small [needles]. And those, as someone said, hopeless ones, who shoot into groin, they hit with [needles from] 5-ml [syringes]. If there are no veins, only tiny veins are left, he would use insulinka [needles from insulin-type syringes].”* (FGD#5, Kulob)

*“I take these 2-ml [syringes], but would attach a longer needle, the blue one or the black, long one, which [syringe] had those? Was it 10-ml? So this kind of needle, I buy them, will tear [the package] off, I throw [the barrel] away, will take its’ needle and will attach it to a 2‐ml [syringe].”* (FGD#6, Kulob)

#### Longer needles are also preferred by those who inject intramuscularly or into deep (central) veins

*“…yes, [intra]muscular, so [the solution] will not go under the skin. Because, if [the needle] is [short], [the solution] will go under the skin.”* (FGD#8, Khorog)

*“It will not work then, it is too short, it will reach only so much, will not reach central veins. So I would use [a needle from a] 2-ml one… it can reach the central veins…].”* (FGD#5, Kulob)

Using longer needles to inject in shallow veins raises the risk of going all the way through the vein and into tissue beneath the vein. In some instances, this may cause bruising and discomfort, and in other instances, it may lead to abscesses or other soft tissue infections. So, most PWID try to avoid using longer needles to inject into shallow veins.

#### Needle gauge

As noted previously, the gauge of a needle is its outside diameter. The diameter of the bore of needles of the same gauge may vary depending on if the *needle* is thin walled or thick walled. Higher gauge (i.e., thinner) needles are preferred for injecting in the smaller veins of the arms or hands. In particular, a needle from the insulin-type syringe (also called a “capillary” needle) is very popular for injecting into thin veins (referred to as capillaries by some of the participants). Some participants mentioned that insulin-type needles can be used for injecting into the surface veins in arms and hands by PWID who have good veins, including those who recently started injecting. PWID often combine the needle from an insulin-type syringe with a barrel from a 2-ml syringe.

*“If someone has visible veins, he would, of course, use insulinka, it has a small needle.”* (FGD#8, Khorog)

*“I have these [insulin-type syringes], it has a thin needle, why do you use them, so the vein does not burst, no blood gets deposited there. [Insulin-type syringes], they have very small needles, so with that, it is a little bit easier with it. You check [if the needle is in the vein], you inject, smoothly. Say, I have no large veins, lost them, so I use insulinka for thin veins.”* (FGD #4, Kulob)

These needles are also preferred by those who want to avoid visible needle puncture marks on their skin, which may reveal that they inject drugs. Injecting with thin needles from insulin-type syringes is also less painful.

*“… it is better with that [needle from an insulin-type syringe], because it will not leave needle traces, needle marks…No bruises.”* (FGD #3, Kulob)

*“…I just hit with insulin-[type syringe], so it does not hurt.”* (FGD#5, Kulob)

Participants reported that PWID often use thicker needles when they are injecting in larger veins. They reported preferring thicker needles if they were injecting more viscous fluids that are difficult to inject through a thin needle. Similarly, participants reported that PWID who have trouble with their blood clotting and clogging the needle prefer thicker needles because they do not clog as easily. Participants in both cities reported these issues as described in the following quotes.

*“Those who use a filter, they can use thin ones, it won’t [get clogged] thanks to filtering. For us it won’t work, because most just shake [drug mixture inside the syringe], so it is not good for us.”* (FGD#5, Kulob)

*“With the brown [hub] needles, say, when you draw the blood in, if you are a bit late, it can get clogged.”* (FGD#4, Khorog)

“[*Needles from insulin-type syringes] or others, 46*^*th*^*, 25*^*th*^*, if you use them, they get clogged, it happens…” (FGD#7, Khorog)*

In general, needles longer than 25 mm tend to be thicker because long thin needles bend too easily. PWID who inject in femoral veins or other deep veins require long thick needles to reach the vein without bending.

#### Needle hub opacity

Several participants reported that they preferred needles with translucent plastic hubs over needles with opaque plastic hubs. With a translucent hub, PWID can see the blood when it is still in the needle hub. With an opaque hub they have to draw blood all of the way into the barrel of the syringe as described in this quote from a participant in Khorog.

*“…you would draw blood back and will not see it, you won’t know if the needle is there [in the vein] or not.”* (FGD#12, Khorog)

Drawing more blood into a syringe increases the possibility that the blood may clot and block the needle. As blood disperses through the drug solution, the solution begins to look like blood. This can be problematic if the needle moves slightly during the injection process, and more blood needs to be drawn into the syringe to confirm that the needle is still in a vein. Opaque needle hubs also make it impossible to tell if there is blood trapped in the hub of the needle after rinsing a syringe with the needle attached. With an opaque needle hub, the only way to determine if blood is still in the needle hub is to remove the needle and look into the hub. These factors may increase the risk of blood borne disease transmission if needles with opaque needle hubs are shared.

#### Syringe barrel capacity

Participants reported that the barrels of 1-ml syringes are long and thin. Using these syringes, it is difficult to operate the plunger with one hand when injecting volumes of fluid much greater than 0.5 ml. A participant from Khorog described the problem as follows:

*“[The insulin-type syringe] is somehow inconvenient, it is small and thin, and it is easier with a 2[ml], with this one, it is easier to handle it with one hand.”* (FGD #12, Khorog)

When injecting into a vein in an arm or hand, it is essential to hold the syringe steady with two fingers and a thumb and operate the plunger with the index finger and the middle finger.

Some homemade drugs require injecting volumes of fluid greater than 2 ml. Also, when PWID are preparing drug solutions for more than one person, they may use 5-ml syringes.

#### Syringe design (permanently attached or detachable needle)

Many PWID reported preferring syringes with detachable needles over syringes with permanently attached needles. In some instances, this was because the maximum barrel capacity of syringes with permanently attached needles in Tajikistan is 1 ml. As noted previously, the barrels of most 1-ml syringes are long and thin. This makes them difficult to manipulate with one hand if a PWID is injecting more than 0.5 ml of fluid, and most PWID in Tajikistan inject at least 1 ml of fluid. In addition to not being available in the appropriate barrel capacity, participants reported several other reasons for preferring detachable needles. If a detachable needle clogs, it is relatively easy to remove it and replace it with another needle. With detachable needles, the needle can be removed, and the tip of the syringe nozzle can be placed directly on the filter. The bore of the syringe nozzle is much larger than the bore of a needle, which allows even high viscosity solutions to be drawn up relatively rapidly. Another potential benefit that was not mentioned is that removing a needle when drawing the drug solution into the syringe virtually eliminates the risk of dulling a needle by accidentally jamming it into the bottom of the mixing vessel (e.g., cooker, spoon).

## Discussion

**Fig. 2:**
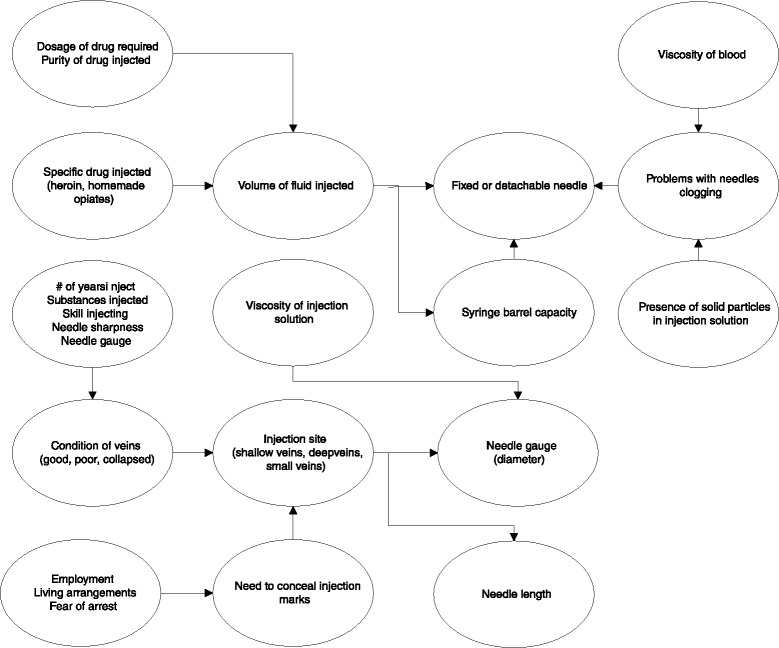
Factors that influence choice of needle gauge, needle length, and syringe barrel capacity and interrelationships among them

A wide variety of interrelated factors influence the characteristics of the needles and syringes that PWID prefer and use. These focus groups provided rich information on the characteristics of the needles and syringes that PWID use and the factors that influence the use of different needles and syringes. We used this information to construct a diagram of the interrelationships among the different factors (Fig. 2).

Factors, such as a preference for high quality needles and syringes or for translucent needle hubs are likely to remain relatively stable over time and are relatively independent of each other. In contrast, the factors that influence choice of needle gauge, needle length, syringe barrel capacity, and syringe design (i.e., permanently attached needle or detachable needles) tend to be interrelated and more variable. Factors such as the condition of a person’s veins are likely to evolve over time. Over the course of an injection career, veins get worse. Other factors, such as syringe barrel capacity may be situational and change depending on the volume of fluid required to prepare a specific drug (e.g., heroin, acetylated opium) or whether the drug solution is prepared for one person or to be divided among two or more people.

It appears that because most PWID in Tajikistan inject heroin almost exclusively, there is less variation in syringe barrel capacity than in cities and countries where PWID inject a variety of drugs [7]. Although most PWID in Khorog and Kulob use 2-ml syringes, some also use 5-ml syringes. According to the participants in these focus groups, almost no PWID in Tajikistan use 1-ml or 10-ml syringes. In a previous study, we found that the dead space in a needle and syringe combination increased as the barrel capacity increased [17]. This association also holds up when low dead space detachable needles are attached to syringe barrels of different capacity. This may make low dead space detachable needles more effective in reducing the probability of HIV and HCV transmission in Tajikistan than in countries such as Ukraine where larger (e.g., 5 ml and 10 ml) syringes are more common [7].

There was much wider variation in terms of needle length and needle gauge. Participants described factors such as injection site, viscosity of the drug solution, and need to conceal injection marks that influenced needle length and gauge. Nevertheless, they reported that they would use whatever needle was available when they were injecting.

These findings suggest that low dead space detachable needles will be acceptable to PWID in these cities as long as they are high quality and available in the appropriate lengths and gauges.

## Limitations

As with most qualitative research, the generalizability of results may be limited. All of the participants in the focus groups reported obtaining some or all of their needles and syringes from NSP. Accordingly, the results may not be generalizable to PWID who do not obtain syringes from NSP. In addition, the focus group participants were not randomly selected from NSP participants, so the findings may not be generalizable to all NSP clients in each city. Nonetheless, the PWID in these focus groups were similar to PWID who participated in integrated bio-behavioral surveys (IBBS) in these cities [1]. The absence of women in the focus groups also limits the generalizability. Although the vast majority of PWID in Tajikistan appear to be male, in some other countries women make up a substantial proportion of PWID. However, to the extent that women inject with men, they are likely to be injecting the same drugs, using the same needles and syringes and facing some of the same injection-related problems [25]. PWID in other cities in Tajikistan may differ from PWID in Kulob and Khorog. So, caution should be used in generalizing these findings to PWID in other cities.

In reporting findings from focus groups and qualitative interviews, the inclusion of direct quotes from participants adds richness to the results. However, these focus groups were conducted in Russian and Tajik and translated into English for analyses. Although the translations and main analyses were performed by researchers who are fluent in all three languages, translated quotes may lose some of the cultural nuances from the original quote.

We did not ask participants if they usually obtained a variety of needles and syringes from the NSP or pharmacy or if they only obtained a single size. It seems likely that many PWID may only ask for the specific needles and syringes that they prefer and use instead of asking for a variety of needles and syringes. In instances where PWID inject together, they inject in different veins/injection sites, but if only one of them has needles and syringes, the other may be forced to use a needle and syringe combination other than he or she prefers.

## Conclusion

Findings from this study suggest that efforts to promote low dead space syringes may fail if they rely solely on 1-ml insulin-type syringes with permanently attached needles. This is especially true in places like Tajikistan where almost all PWID use 2-ml or larger syringes and prefer detachable needles. However, low dead space detachable needles may provide an acceptable alternative in these areas, which could greatly expand the impact of low dead space needles and syringes on reducing HIV and HCV among PWID globally.

It is important for policy makers and those responsible for NSP purchasing decisions to ensure that NSP distribute high quality needles and syringes with attributes (e.g., barrel capacity, needle length, needle gauge) that are acceptable to NSP clients. Prior to implementing the WHO recommendation to offer their clients low dead space needles and syringes, NSP need to ascertain the needs of their clients and offer low dead space equipment that will be acceptable to their clients. To ensure uptake of low dead space equipment, NSP may also have to provide educational and motivational materials regarding the benefits of switching to low dead space needles and syringes.

## References

[1] Latypov A, Otiashvili D, Zule W (2014). Drug scene, drug use and drug-related health consequences and responses in Kulob and Khorog, Tajikistan. Int J Drug Policy.

[2] Walsh N, Maher L (2013). HIV and HCV among people who inject drugs in Central Asia. Drug Alcohol Depend.

[3] Bailey SL, Ouellet LJ, Mackesy-Amiti ME, Golub ET, Hagan H, Hudson SM, Latka MH, Gao W, Garfein RS (2007). Perceived risk, peer influences, and injection partner type predict receptive syringe sharing among young adult injection drug users in five U.S. cities. Drug and alcohol dependence.

[4] Pouget ER, Deren S, Fuller CM, Blaney S, McMahon JM, Kang SY, Tortu S, Andia JF, Des Jarlais DC, Vlahov D (2005). Receptive syringe sharing among injection drug users in Harlem and the Bronx during the New York State Expanded Syringe Access Demonstration Program. Journal of acquired immune deficiency syndromes (1999).

[5] Grund JP, Friedman SR, Stern LS, Jose B, Neaigus A, Curtis R, Des Jarlais DC (1996). Syringe-mediated drug sharing among injecting drug users: patterns, social context and implications for transmission of bloodborne pathogens. Soc Sci Med (1982).

[6] Koester S, Booth RE, Zhang Y (1996). The prevalence of additional injection-related HIV risk behaviors among injection drug users. J Acquir Immune Defic Syndr Hum Retrovirol.

[7] Ibragimov U, Latypov A (2012). Needle and syringe types used by people who inject drugs in Eastern Europe and central Asia: key findings from a rapid situation assessment.

[8] Gaughwin MD, Gowans E, Ali R, Burrell C (1991). Bloody needles: the volumes of blood transferred in simulations of needlestick injuries and shared use of syringes for injection of intravenous drugs. AIDS (London, England).

[9] Zule WA, Ticknor-Stellato KM, Desmond DP, Vogtsberger KN (1997). Evaluation of needle and syringe combinations. J Acquir Immune Defic Syndr Hum Retrovirol.

[10] Vickerman P, Martin NK, Hickman M (2013). Could low dead-space syringes really reduce HIV transmission to low levels?. Int J Drug Policy.

[11] Zule WA, Cross HE, Stover J, Pretorius C (2013). Are major reductions in new HIV infections possible with people who inject drugs? The case for low dead-space syringes in highly affected countries. Int J Drug Policy.

[12] Abdala N, Stephens PC, Griffith BP, Heimer R (1999). Survival of HIV-1 in syringes. J Acquir Immune Defic Syndr Hum Retrovirol.

[13] Paintsil E, He H, Peters C, Lindenbach BD, Heimer R (2010). Survival of hepatitis C virus in syringes: implication for transmission among injection drug users. J Infect Dis.

[14] Bobashev GV, Zule WA (2010). Modeling the effect of high dead-space syringes on the human immunodeficiency virus (HIV) epidemic among injecting drug users. Addiction.

[15] Walsh N, Verster A, Rodolph M, Akl EA (2014). WHO guidance on the prevention of viral hepatitis B and C among people who inject drugs. Int J Drug Policy.

[16] Senbanjo R, Strang J (2011). The needle and the damage done: clinical and behavioural markers of severe femoral vein damage among groin injectors. Drug Alcohol Depend.

[17] Zule W, Pande P, Bobashev G, Coomes C, Des Jarlais D, Friedman S, Gyarmathy V, Otiashili D, Poulton W (2010). Variations in needles and syringes used by injecting drug users. In: NIDA International Forum.

[18] Bernard HR (2000). Social research methods : qualitative and quantitative approaches.

[19] Ishak N, Bakar A (2012). Qualitative data management and analysis using NVivo: an approach used to examine leadership qualities among student leaders. Educ Res J.

[20] Hruschka DJ, Schwartz D, John DCS, Picone-Decaro E, Jenkins RA, Carey JW (2004). Reliability in coding open-ended data: Lessons learned from HIV behavioral research. Field Methods.

[21] Fleiss JL (1971). Measuring nominal scale agreement among many raters. Psychol Bull.

[22] Miles Matthew B, Huberman AM, Saldana J (2014). Qualitative data analysis, A methods sourcebook edisi ketiga.

[23] Landis JR, Koch GG (1977). The measurement of observer agreement for categorical data. Biometrics.

[24] Higgs P, Dwyer R, Duong D, Thach ML, Hellard M, Power R, Maher L (2009). Heroin-gel capsule cocktails and groin injecting practices among ethnic Vietnamese in Melbourne. Australia. Australia Int J Drug Policy..

[25] Syvertsen JL, Robertson AM, Strathdee SA, Martinez G, Rangel MG, Wagner KD (2014). Rethinking risk: gender and injection drug-related HIV risk among female sex workers and their non-commercial partners along the Mexico-U.S. border. Int J Drug Policy.

